# Molecular characteristics of a coxsackievirus A12 strain in Zhejiang of China, 2019

**DOI:** 10.1186/s12985-022-01892-1

**Published:** 2022-10-12

**Authors:** Linjie Hu, Lu Zhou, Pingping Wang, Hairenguli Maimaiti, Yihan Lu

**Affiliations:** 1 Department of Epidemiology, Ministry of Education Key Laboratory of Public Health Safety, School of Public Health, Fudan University, Fosun Tower, 131 Dong An Road, Shanghai, 200032 China; 2 Pujiang Center for Disease Control and Prevention, Jinhua, 321000 Zhejiang China

**Keywords:** Enterovirus, Coxsackievirus A12, Recombination, Phylogenetic analysis

## Abstract

**Background:**

Enterovirus A (EV-A), such as enterovirus A71 (EV-A71), generally causes hand, foot, and mouth disease (HFMD). However, limited studies focused on uncommon enterovirus serotypes such as coxsackievirus A12 (CV-A12). This study aimed to provide evidence to determine the molecular characteristics of a CV-A12 strain isolated in Zhejiang province, China.

**Methods:**

In routine surveillance of HFMD, we identified a child case with CV-A12 infection in 2019 in Zhejiang province, China. Enterovirus was examined by using real-time reverse transcription-PCR (qRT-PCR). A partial VP1 sequence was amplified to determine the serotype, and then a full-length CV-A12 genome was sequenced. Nucleotide and amino acid similarity was calculated with those CV-A12 strains available in GenBank. Recombination was detected using RDP 4 and SimPlot. Furthermore, phylogenetic analysis was conducted by using BEAST 1.10, and protein modeling was performed with I-TASSER webserver.

**Results:**

A full-length CV-A12 genome PJ201984 was isolated in a Chinese child with HFMD. The similarities with complete coding sequences of the CV-A12 strains in GenBank ranged between 79.3–100% (nucleotide) and 94.4–100% (amino acid), whereas it was 88.7–100.0% (nucleotide) and 97.2–100% (amino acid) when excluding the CV-A12 prototype strain Texas-12. In PJ201984, amino acid variations were more divergent in P2 and P3 regions than those in P1; the majority of those variations in VP1 (13/15) and VP4 (7/8) were similar to those documented in recently isolated CV-A12 strains in China. Furthermore, recombination was identified in P2 region, which involved a CV-A5 strain collected in China. Phylogenetic analysis revealed that PJ201984 clustered together with multiple CV-A12 strains isolated in China and the Netherlands during 2013–2018, as compared to another cluster consisting of CV-A12 strains in China and France during 2009–2015. Additionally, protein models of VP1 and VP4 in PJ201984 were well predicted to be similar to VP1 protein of EV-A71 and VP4 protein of coxsackievirus A21, respectively.

**Conclusions:**

The full-length CV-A12 genome was characterized to have common recombination in P2 region and be phylogenetically related to those CV-A12 strains isolated in recent years, suggesting a continual spread in China. It warrants strengthening the routine surveillance for uncommon enterovirus serotypes, particularly on possible recombination and variation.

**Supplementary Information:**

The online version contains supplementary material available at 10.1186/s12985-022-01892-1.

## Introduction

Enterovirus (EV) is a positive-sense single-stranded picornaviridae virus associated with multiple human and mammalian diseases, including hand, foot, and mouth disease (HFMD), herpangina, paralytic disease, aseptic meningitis, and myocarditis [1]. So far, more than one hundred enterovirus serotypes have been identified, of which 25 were classified as enterovirus A (EV-A) [2]. In EV-A, coxsackievirus A12 (CV-A12) has been rarely detected in routine surveillance of enterovirus [3–7], as compared to other common EV-A such as enterovirus A71 (EV-A71) or coxsackievirus A16 (CV-A16). However, CV-A12 was found to be one of the most common pathogens (22/50, 44.0%) among HFMD cases unrelated to EV-A71 or CV-A16 in the 2008–2011 surveillance in Shandong, China [8]. A Chinese national surveillance of HFMD has been established since 2008. Currently, EV-A71 and CV-A16 have been included as major pathogens in the routine surveillance, and coxsackievirus A6 (CV-A6) and A10 (CV-A10) included in the surveillance in some regions; however, other enterovirus serotypes not been routinely monitored [9, 10]. Thus, it remains unclear if CV-A12 is commonly circulated in China. Furthermore, CV-A12 was also documented to be associated with HFMD and herpangina in Thailand [4] and prevalent in Nigeria [11], raising a further public health concern.

With the introduction of EV-A71 vaccine in China’s mainland, distribution of EV-A serotypes has changed in recent years [12, 13]. However, limited studies focused on uncommon enterovirus serotypes causing HFMD, and relatively few sequences were submitted to the GenBank database, which may lead to a lack of knowledge of these serotypes. This study aimed to characterize a full-length CV-A12 genome, which is an uncommon EV-A serotype contributable to HFMD, and further provide evidence to comprehensively understand the pathogens of HFMD.

## Methods

### Study design

In 2019, a total of 102 throat swabs were randomly collected in the routine surveillance from children with HFMD by Pujiang Center for Disease Control and Prevention (CDC) in Zhejiang province, China. Case definition of HFMD referred to Chinese guidelines for the diagnosis and treatment of HFMD (2018 edition) [9]. All the cases had similar mild symptoms, including rash on hand, foot and buttocks with a fever < 38 °C or no fever. Laboratory examination was performed by the CDC laboratory. A total of 63 specimens were successfully serotyped, including EV-A71 (n = 3), CV-A16 (n = 40), CV-A6 (n = 17), and CV-A10 (n = 3), by using qPCR. In addition, 21 were unserotyped (positive for universal enterovirus primers, while not EV-A71, CV-A16, CV-A6 or CV-A10) and 18 were negative.

In this study, we retrospectively determined the serotypes for those 21 unserotyped specimens. Of them, we identified CV-A16 (n = 1), CV-A6 (n = 13), CV-A10 (n = 1), uncommon EV-A serotypes such as CV-A12 (n = 1), CV-A9 (n = 1), CV-A2 (n = 1) and CV-A4 (n = 1), in addition to two negative specimens. The case with CV-A12 had mild rash on hand and foot, while had no fever. Furthermore, we obtained the full-length CV-A12 genome and conducted bioinformatics analysis.

### Laboratory examination

In the study, we placed 200 μL of throat swab lyophilization solution into the automatic nucleic acid extractor (BioGerm, Shanghai, China). Viral RNA extraction was performed using a fully automated nucleic acid purification system (BioGerm, Shanghai, China).

The extracted RNA was then examined using Enterovirus Universal RNA Detection Kit (BioGerm, Shanghai, China) by real-time reverse transcription-PCR (qRT-PCR) conducted on 7500 fast system (Applied Biosystems, Massachusetts, USA). Thermal profiles were 50 °C for 10 min reverse transcription, 95 °C for 5 min pre-denaturation and 95 °C for 10 s, and 55 °C for 40 s denaturation for 40 cycles. We used the FAM channel for examination of enterovirus. The interpretation of qRT-PCR was judged based on the cycle threshold (Ct) value. Specimens with Ct ≤ 35 were judged as positive, and those with Ct ≥ 38 were negative. For the specimens with Ct values ranging 35–38, repeat testing was conducted.

### Serotyping and full-length sequencing

We amplified a partial VP1 sequence for enterovirus serotyping, using the OneStep RT-PCR Kit (QIAGEN, Germany). Nested PCR was performed, including the first round with primers OL68-1 and MD90 and the second round with primers OL68-1 and EVP4, producing a 657 bp partial VP1 sequence (Table 1). PCR products were electrophoresed and then serotyped using the enterovirus typing tool [14].

**Table 1:** Primers for amplification of a 657 bp partial VP1 sequence for enterovirus serotyping and full-length coxsackievirus A12 (CV-A12) genome

Primer	Primer sequence
OL68-1	5′-GGTAAYTTCCACCACCANCC-3′
MD90	5′-CCTCCGGCCCCTGAATGCGGCTAAT-3′
EVP4	5′-CCTCCGGCCCCTGAATGCGGCTAAT-3′
CA12_F1	TTGTACCAACTCACAGGGC
CA12_R1	GARTTGCCAACAGTYAGTTG
CA12_F2	AGCTATTGGATTGGCCATCC
CA12_R2	GTTGGTCACCTCTCCAGG
CA12_F3	ACAGCCATACCAATCACTAT
CA12_R3	GTRAAGATCTCYAGCTTGCG
CA12_F4	TTACAAGCAGCAGARACAGG
CA12_R4	CTCTGGATACTGCRTCAGTG
CA12_F5	CAGACTGGAGTGTACTATTG
CA12_R5	TCACTTCTATRTCACAGTCC
CA12_F6	GTGGTCACAGTYATGGATG
CA12_R6	TCACTGGCRAAGTAGCTTC
CA12_F7	ACTAAGTTCATCCCAGAGA
CA12_R7	GCGTCATACCCTGAGTAATC
CA12_F8	TGGACAAGTATGGTTTGG
CA12_R8	GCTACTCTGGTTATAACAAA

Moreover, a full-length CV-A12 genome PJ201984 was sequenced (BioGerm, Shanghai, China) by using a group of CV-A12 primers (Table 1). SeqMan in the Lasergene v7.0 (DNASTAR, the United States) package was used to splice the sequencing fragments. The enterovirus serotype was further confirmed using the Basic Local Alignment Search Tool (BLAST) [15]. The full-length CV-A12 genome PJ201984 has been deposited in GenBank under the accession number OM638431.

### Datasets of sequences

Complete coding sequences (CDS) of CV-A12 and other EV-A prototype strains were searched in the GenBank database, of which those sequences with high similarity to PJ201984 were retrieved using the BLAST tool to construct the dataset 1 of complete CDS (Additional file 1: Table S1). This dataset contained two subsets. The first subset was prototype subset, containing all EV-A serotype prototype strains and all CV-A12 strains. The second subset was BLAST subset, including all the sequences retrieved using the BLAST and the CV-A12 prototype strain Texas-12. In the study, we used the first subset for similarity analysis and the second one for recombination analysis.

Furthermore, all VP1 sequences of CV-A12 in the GenBank database were retrieved and globally compared using the FFT-NS-2 strategy based on MAFFT v7.311 [16]. Considering the lengths of those VP1 sequences were diverse, a 270 bp partial sequence was accordingly determined for further analysis, which ensured the inclusion of the maximum number of CV-A12 sequences to retain sufficient spatial and temporal information of CV-A12. A random screening was performed using cd-hit web server [17] with a cut-off value of 99.5% for random screening to build the dataset 2 of 270 bp partial VP1 sequences (Additional file 1: Table S2). In addition, a dataset 3 of full-length VP1 sequences was prepared for phylogenetic analysis (Additional file 1: Table S3).

### Similarity and variation analysis

Sequence alignment was performed using MAFFT v7.311, and then clipped using Molecular Evolutionary Genetics Analysis (MEGA) X [18]. The sequences were compared using MegAlign in Lasergene v7.0 package for calculation of similarity and divergence. Heatmaps were illustrated using R pheatmap package [19]. Furthermore, nucleotide and amino acid residue variation was identified using the ClustalW aligning method. Transition/transversion (ti/tv) ratio was calculated using maximum likelihood method. In addition, the twenty amino acids were divided into four groups: aliphatic amino acids (G, A, V, L, I); hydrophilic amino acids (S, T, C, M, N, Q); charged amino acids (D, E, K, R); aromatic and heterocyclic amino acids (F, Y, W, H, P). Similar amino acid substitutions were classified as the substitution within the same groups, and different substitutions were those across the groups.

### Detection of recombination

Recombinant analysis was performed in the dataset 1 of complete CDS, including CV-A12 and other EV-A prototype strains, using Recombination Detection Program v4 (RDP4) [20] and SimPlot v3.5.1 [21]. We detected the beginning and ending breakpoints using the bootscan algorithm and illustrated the RDP plot and simplot. In the bootscan analysis, we constructed the Neighbor-Joining (NJ) trees to determine the phylogenetic relationship of sequences, using 1000 bootstrap replicates, Kimura-2 model, and 95% cut-off percentage for “calculate binomial P-value”, according to the recommendations in the RDP4 instruction manual.

### Phylogenetic analysis

The dataset 2 of 270 bp partial VP1 sequences and the dataset 3 of full-length VP1 sequences were detected by using TempEst [22] for temporal signal, with plotting root-to-tip graph and calculating temporal correlation coefficient. The optimal nucleotide substitution model was determined using MEGA X after confirming the temporal signal. Then the maximum clade credibility (MCC) phylogenetic tree was reconstructed by using Bayesian Evolutionary Analysis Sampling Trees (BEAST) v1.10 [23]. The TN93 + G [5] model was selected, with a lognormal uncorrelated relaxed clock as clock model and exponential growth as tree prior. We utilized the traits to characterize the spatial and temporal information of the CV-A12 VP1 sequences. The procedure was determined to be 10,000,000 states and 1000 steps for MCMC under 10% burn-in to achieve the convergence. FigTree v1.4.4 [24] was used to visualize the tree.

### Protein modeling of VP1 and VP4

Protein model prediction of VP1 and VP4 within PJ201984 was performed using the I-TASSER web server [25, 26], and the ResQ method was used to calculate B-factor [27]. The B-factor is a value indicating the intrinsic thermal mobility of amino acid residues/atoms in the protein, and the value below zero indicates the predicted relative stability of the structure in the segment. Editing of protein structure was performed using the PyMol program (Schrödinger Inc., California, USA).

## Results

### Nucleotide and amino acid similarity

**Fig. 1:**
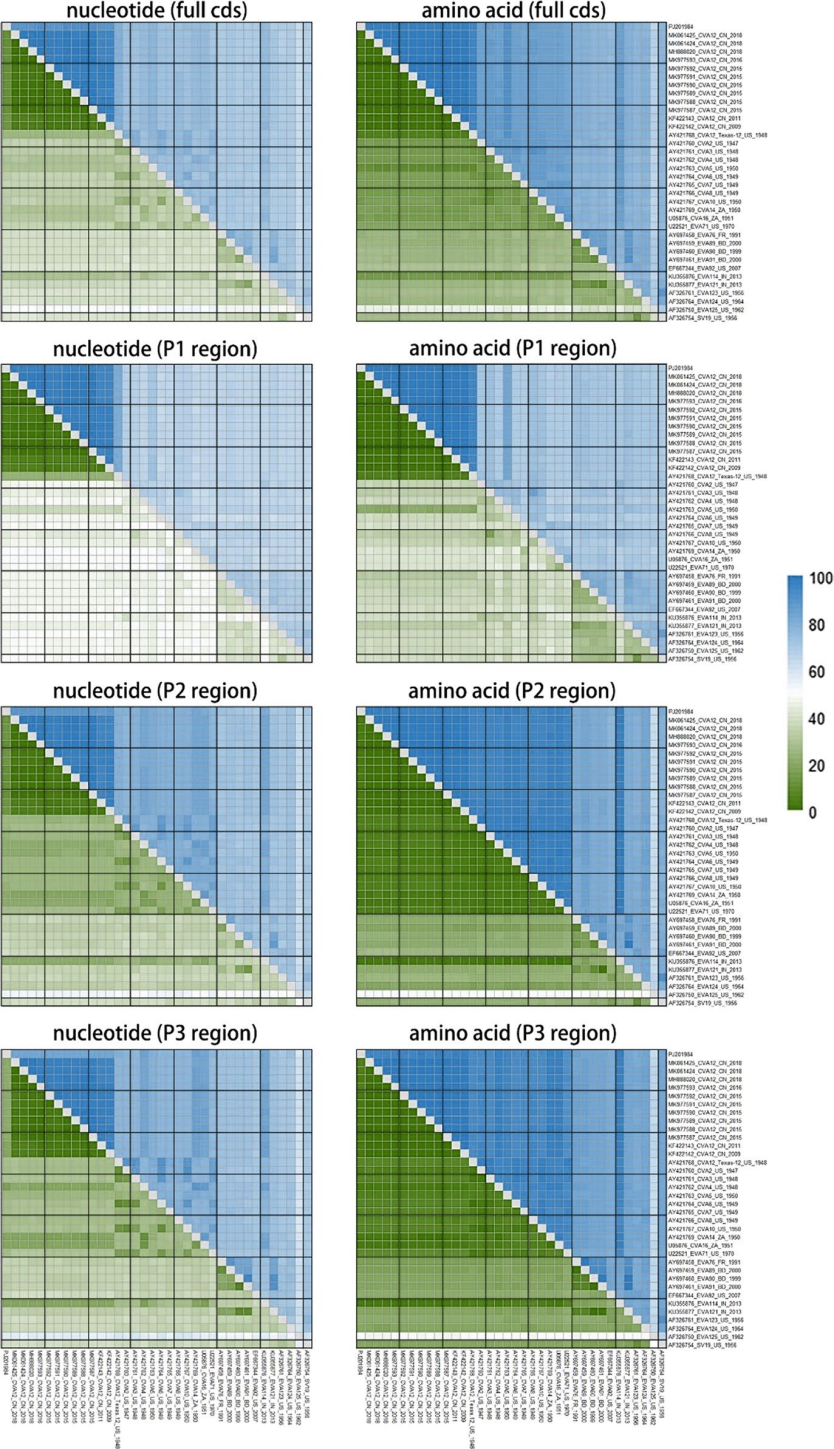
Similarity and divergence heatmaps of full-length coxsackievirus A12 (CV-A12) sequences and other enterovirus A (EV-A) prototype strains (prototype subset within the dataset 1). The percentage of similarity and divergence was shown in the upper right triangle and lower left triangle, respectively. The full-length CV-A12 genome PJ201984 isolated in the study was listed as the first sequence, followed by 13 full-length CV-A12 sequences and 22 other EV-A prototype strains retrieved in GenBank

The full-length CV-A12 genome PJ201984 had high similarities to the CV-A12 strains available in GenBank, ranging between 79.3–100% (nucleotide) and 94.4–100% (amino acid) (Table 2). As the CV-A12 prototype strain Texas-12 was phylogenetically distant from other CV-A12 genomes recently isolated in China, which may remarkably influence the similarity. Thus, we presented the similarity including and excluding Texas-12 for comparison. They increased to 88.7–100% (nucleotide) and 97.2–100% (amino acid) when excluding Texas-12 that was isolated in 1948 in the United States. Furthermore, we found that the full-length nucleotide sequences differed by EV-A serotypes, while the amino acid sequences were relatively similar (Fig. 1).

**Table 2:** Pairwise similarity of full-length coxsackievirus A12 (CV-A12) genome PJ201984 with other CV-A12 sequences in GenBank by complete and partial coding sequences (CDS)

	All CV-A12 sequences		CV-A12 sequences (CV-A12 prototype strain Texas-12 excluded)	
	Nucleotide (%)	Amino acid (%)	Nucleotide (%)	Amino acid (%)
Complete CDS	79.3–100.0	94.4–100.0	88.7–100.0	97.2–100.0
P1	80.7–99.9	94.6–100.0	94.1–99.9	98.5–100.0
P2	78.9–100.0	95.2–100.0	87.6–100.0	97.1–100.0
P3	77.0–100.0	93.1–100.0	82.4–100.0	95.5–99.9

### Nucleotide and amino acid variation

**Fig. 2:**
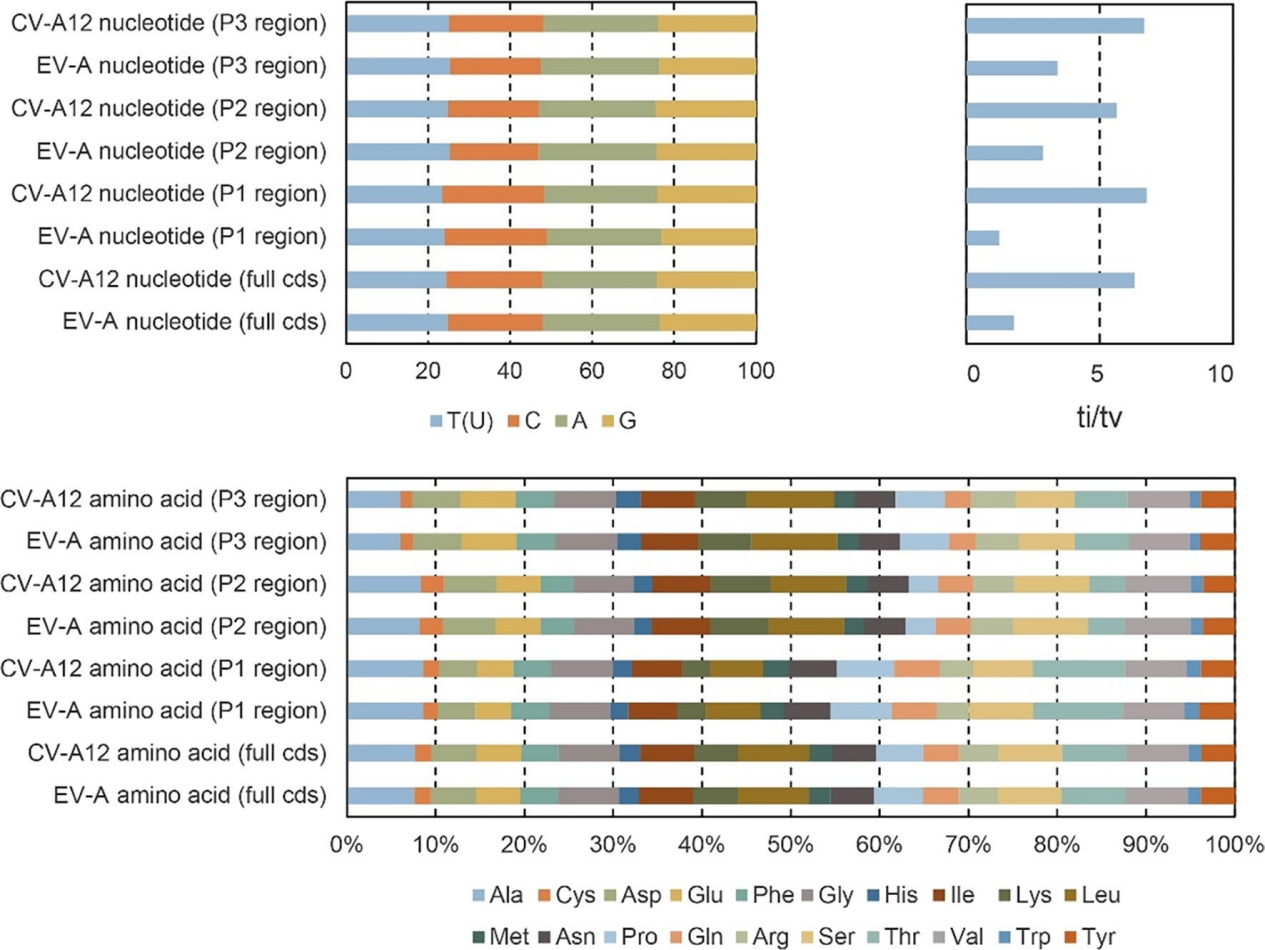
Nucleotide and amino acid composition and transition/transversion (ti/tv) ratio, stratified by all full-length coxsackievirus A12 (CV-A12) strains and enterovirus A (EV-A) strains (including CV-A12) listed in the dataset 1

The nucleotide and amino acid compositions of complete CDS, P1, P2, and P3 coding regions were similar between all CV-A12 strains and all EV-A strains in the dataset 1 (Fig. 2). However, the ti/tv ratio was remarkably higher in the CV-A12 strains than that in other EV-A strains, regardless of complete CDS, P1, P2, or P3.

**Fig. 3:**
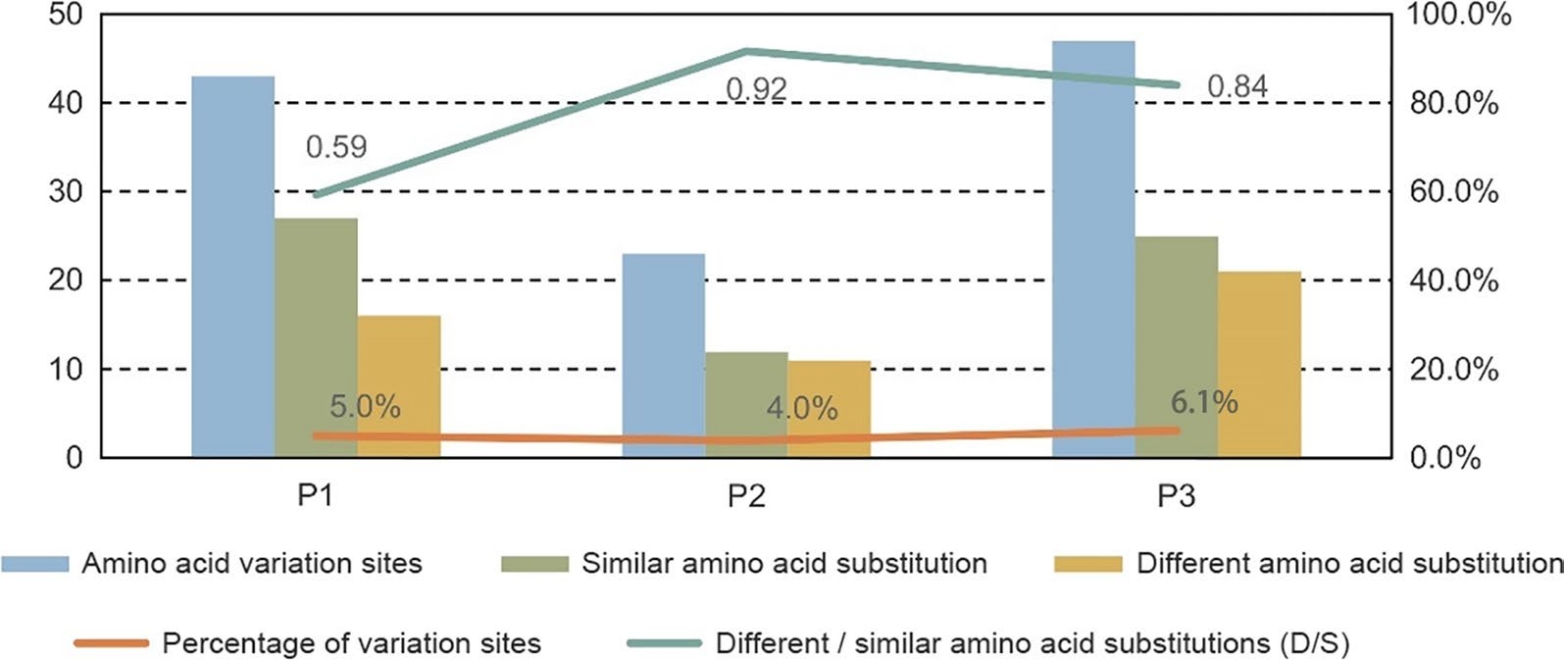
Number and percentage of amino acid variation sites and substitution type of the full-length coxsackievirus A12 (CV-A12) genome PJ201984 isolated in the study, as compared to the CV-A12 prototype strain Texas-12. Similar amino acid substitutions were classified as the substitutions within the same amino acid groups, including aliphatic amino acids (G, A, V, L, I), hydrophilic amino acids (S, T, C, M, N, Q), charged amino acids (D, E, K, R), and aromatic and heterocyclic amino acids (F, Y, W, H, P). Different substitutions were classified as those across the groups

We further compared the amino acid variation sites in PJ201984 with the CV-A12 prototype strain Texas-12. A total of 43 sites (43/860, 5.0%) were found in P1 region, including 27 similar amino acid substitutions and 16 different amino acid substitutions (Fig. 3). Furthermore, 23 sites (23/578, 4.0%) were found in P2 region, including 12 similar and 11 different amino acid substitutions; 46 sites (46/754, 6.1%) were found in P3 region, including 25 similar and 21 different amino acid substitutions.

### Recombination event

**Fig. 4:**
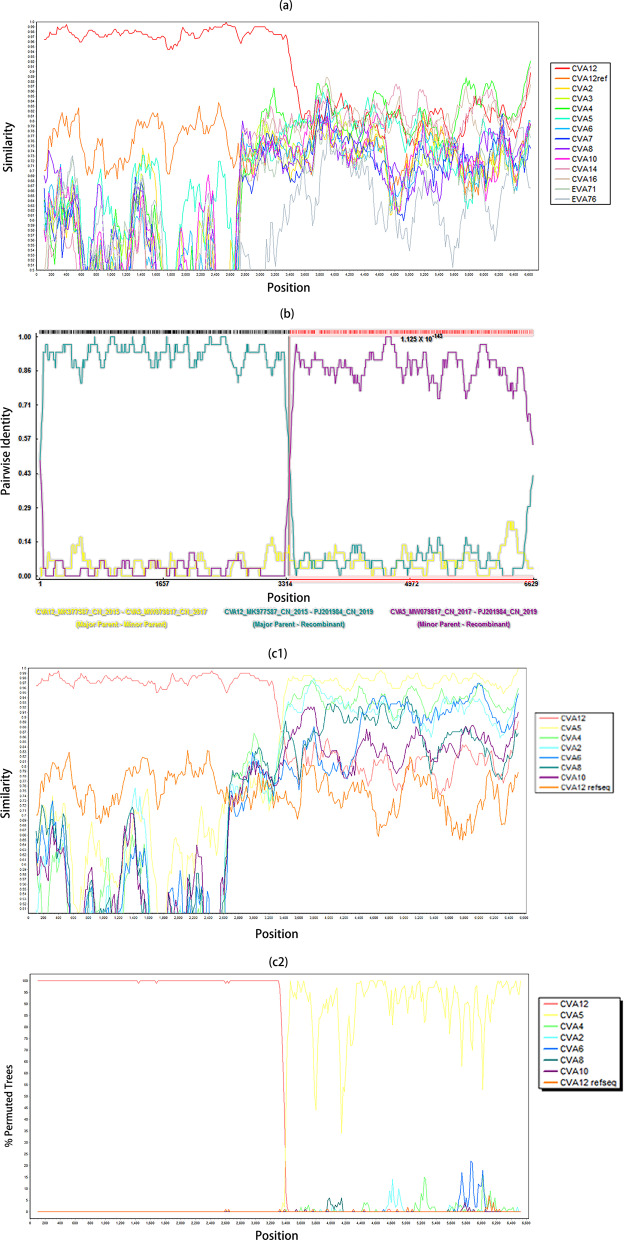
**a** Simplot of general recombination identified in P2 region of the full-length coxsackievirus A12 (CV-A12) genome PJ201984 isolated in the study, as compared to CV-A12 strains and other enterovirus A (EV-A) prototype strains (prototype subset within the dataset 1), by using SimPlot. In the legend, “CVA12” indicated the combined group of all CV-A12 sequences retrieved in GenBank except CV-A12 prototype strain Texas-12; “CVA12ref” was Texas-12. **b** Recombination detection plot of PJ201984 by using RDP4. The breakpoint was 3302 nucleotide site in the complete coding sequence of PJ201984. **c** Simplot (1) and bootscan plot (2) performed in BLAST subset within the dataset 1, by using SimPlot. The bootscan analysis predicted that the recombination occurred between PJ201984 and a coxsackievirus A5 (CV-A5) strain isolated in 2017 in China (GenBank accession number MW079817). In the legend, “CVA12” indicated the combined group of all CV-A12 sequences retrieved in GenBank except CV-A12 prototype strain Texas-12; “CVA12ref” was Texas-12

The P1 region was conserved in PJ201984. In contrast, the P2 and P3 regions in PJ201984 were highly divergent with CV-A12 strains or other EV-A prototype strains (Fig. 4a). Furthermore, the recombination breakpoint was identified to locate at the 3302-nucleotide site in the complete CDS of PJ201984 (Fig. 4b). PJ201984 was most similar to a CV-A12 sequence isolated in 2015 in China (GenBank accession number MK977587) that was considered a major parent and recombined with a CV-A5 isolated in 2017 in China (GenBank accession number MW079817) that was considered a minor parent. The possible recombination between CV-A12 and CV-A5 in the P2 region was further verified using bootscan analysis (Fig. 4c1, c2).

### Phylogenetic analysis

**Fig. 5:**
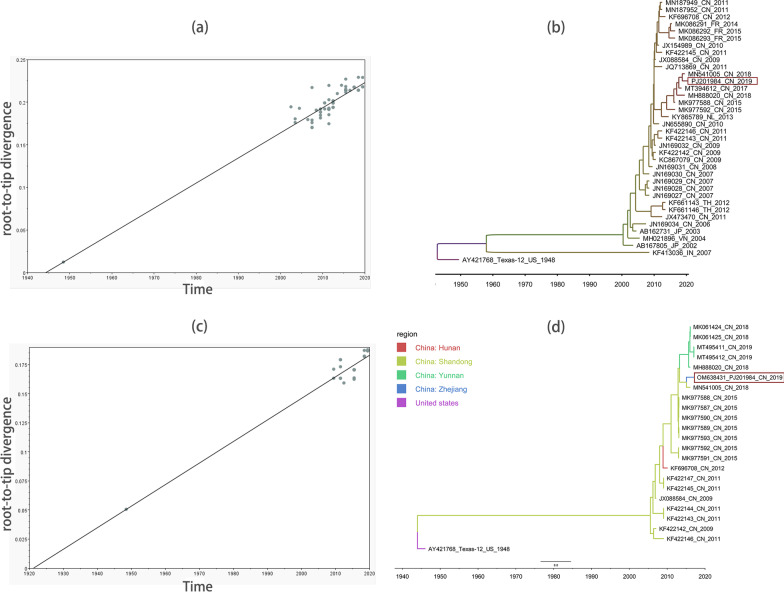
**a** Root-to-tip divergence of 270 bp partial coxsackievirus A12 (CV-A12) VP1 sequences with correlation coefficient of 0.9449, R squared of 0.8929, and X-Intercept of 1944.3109, by using TempEst. **b** The maximum clade credibility (MCC) phylogenetic tree reconstructed with 270 bp partial VP1 sequences by using BEAST. The year of CV-A12 divergence was determined to be 1937.802, with each estimate sample size > 200. **c** Root-to-tip divergence of full-length CV-A12 VP1 sequences with correlation coefficient of 0.9690, R squared of 0.9389, and X-Intercept of 1921.1878, by using TempEst. **d** The MCC phylogenetic tree reconstructed with full-length VP1 sequences by using BEAST. The year of CV-A12 divergence was determined to be 1944.196, with each estimate sample size > 200

According to the detection of temporal signal, all 270 bp partial VP1 sequences (Fig. 5a) and those full-length VP1 sequences (Fig. 5c) had temporal association on the collection date. Phylogenetic analysis of 270 bp VP1 sequences revealed that PJ201984 clustered together with multiple CV-A12 strains isolated in China and the Netherlands during 2013–2018, as compared to another cluster consisting of CV-A12 strains in China and France during 2009–2015 (Fig. 5b). It might suggest a continual expansion of CV-A12 population in China, as compared to previously circulation of CV-A12 in some Asian countries, including India, Japan, Vietnam, and Thailand. Moreover, we classified the collection Chinese regions in the phylogenetic analysis of full-length VP1 sequences (Fig. 5d). It revealed a detailed distribution of CV-A12 strains isolated in China, among which those strains in Shandong were predominant.

### Molecular characteristics of VP1 and VP4

In this study, we determined the amino acid variation in VP1 and VP4 coding regions among PJ201984, the CV-A12 prototype strain Texas-12, and those CV-A12 strains isolated in China during 2009–2018 (Table 3). There were 15 sites of amino acid substitutions in VP1 while eight in VP4. Majority of amino acid variations in VP1 (13/15) and VP4 (7/8) in PJ201984 were similar to those CV-A12 strains recently isolated in China, suggesting a substantial consistency. However, there were three different substitutions of amino acids between PJ201984 and the consensus strain of CV-A12 strains isolated recently in VP1 and VP4 regions (bolded rows in Table 3).

**Table 3:** Amino acid variation sites in VP1 and VP4 of full-length coxsackievirus A12 (CV-A12) PJ201984, CV-A12 prototype strain Texas-12, and consensus CV-A12 sequence

Protein	Variation sites	PJ201984	Texas-12	Consensus CV-A12*
VP1	**3**	**A**	**T**	**T**
	17	N	S	N
	**27**	**S**	**T**	**T**
	35	N	T	N
	61	S	N	S
	71	K	R	K
	91	V	I	V
	93	V	I	V
	143	N	S	N
	149	L	M	L
	177	I	V	I
	233	M	I	M
	247	V	I	V
	290	T	A	T
	294	A	S	A
VP4	9	K	R	K
	10	S	T	S
	17	T	N	T
	18	F	I	F
	20	S	T	S
	51	S	N	S
	**57**	**M**	**I**	**I**
	64	T	V	T

*The consensus CV-A12 sequence was defined as a combination of the majority amino acid in each site, generated by MegAlign

The bolded rows indicated the differences between the PJ201984 and the consensus CV-A12 sequence

**Fig. 6:**
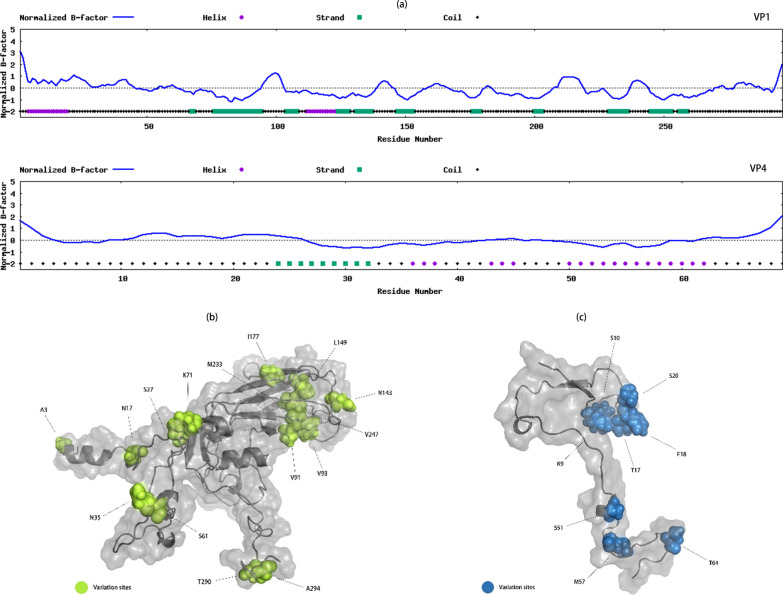
**a** Secondary structure and B-factor plot of VP1 (upper) and VP4 (down) of the full-length coxsackievirus A12 (CV-A12) genome PJ201984 isolated in the study. In addition, the purple sites were located in helix structures, and the green sites in strand structures. **b** Modeling of VP1 protein of PJ201984. **c** Modeling of VP4 protein of PJ201984

We further found that the prediction of VP1 and VP4 protein secondary structures for PJ201984 had low B-factors (Fig. 6a). Then the structures of VP1 and VP4 proteins and amino acid variation sites were illustrated (Fig. 6b, c). The best predicted models of VP1 and VP4 protein obtained a C-score of 0.72 and − 0.41, and TM-score of 0.81 ± 0.09 and 0.66 ± 0.13, respectively. The most similar protein structure to VP1 protein of PJ201984 was that in 3vbfA protein (a VP1 protein of EV-A71) in the PDB database with an identity of 0.536, and the one most similar to VP4 protein of PJ201984 was 1z7sA protein (a VP4 protein of coxsackievirus A21) with an identity of 0.612.

## Discussion

In this study, we isolated a full-length CV-A12 genome PJ201984 in a child with HFMD from the routine HFMD surveillance in Zhejiang province of China in 2019. CV-A12 is an uncommon serotype among the enteroviruses contributable to HFMD among children. Similarity analysis showed serotype-specificity, mainly in the coding region of precursor protein P1, which was consistent with previous literature showing that the structural protein-coding regions were relatively conserved in enteroviruses [28]. In contrast, the differences among serotypes were not evident in P2 and P3 regions, expected for simian enteroviruses. Additionally, nucleotide and amino acid compositions across all CV-A12 strains and all EV-A strains were similar, which might be attributable to common factors associated with viral replication for all enterovirus serotypes. However, the ti/tv differed significantly, suggesting a substantial difference across the serotypes.

We further detected general recombination in P2 regions of PJ201984. It was consistent with the previous findings that recombination was likely to occur in P2 or P3 regions, other than conserved P1 region [29–31]. Moreover, PJ201984 was identified to recombine with a CV-A5 strain that was isolated in Hubei province of China in 2017. In our study, a CV-A12 strain isolated in 2015 (MK977587) and a CV-A5 strain isolated in 2017 (MW079817) were considered as the major and minor parents to the strain PJ201984. These two strains were both collected in China, suggesting that co-circulation of diverse enterovirus serotypes may have resulted in recombination. The recombination event probably occurred in the 2A region of PJ201984, which was common in other enterovirus serotypes in previous findings [32]. In PJ201984, P2 region had the lowest variation, whereas had the highest different amino acid substitution. The findings revealed that the variations in P2 region might result in changing protein properties, as documented elsewhere [33]. Similarly, P3 region tended to be unstable because of the highest variation.

In combination of the similarity plot and the temporal signal detection of the partial and full-length CV-A12 VP1 sequences, we revealed that the CV-A12 sequences included in the study were phylogenetically distant from the CV-A12 prototype strain Texas-12 isolated in 1948. The similarity among the CV-A12 VP1 sequences varied along with the time interval of collection date, suggesting a negative correlation between the time interval and similarity. Meanwhile, according to the previous genotyping study of CV-A12 [8], we found that the CV-A12 sequences isolated in recent years (after 2011) showed a tendency to develop into new subtypes or clusters, as shown in the MCC phylogenetic tree. It indicated that CV-A12 became locally circulating, despite the limited number of isolations, which might lead to increasing divergence compared with CV-A12 prototype strain. Therefore, it warrants strengthening the routine surveillance of uncommon enterovirus serotypes, such as CV-A12, especially for cross-regional transmission.

Based on the protein model prediction, we found that the variations in VP1 and VP4 regions of PJ201984 were not the predicted binding-related and neutralization sites. So far, there has been no evidence revealing that certain variations in VP1 and VP4 of CV-A12 would change viral infectivity and pathogenesis. However, variations in these regions of other enterovirus serotypes may enhance or reduce viral invasion and pathogenicity. For instance, glutamic acid (E) at VP1-145 of EV-A71 was virulent to neonatal mice and transgenic mice expressing human scavenger receptor B2 [34]. In contrast, the VP1-A289T variant of EV-A71 weakened the binding capacity between VP1 and vimentin, and then reduced the infection towards the central nervous system [35]. Furthermore, some variations that occurred on the virulent variant (CB4-V) of coxsackievirus B4 increased the antigenicity, potentially inducing more severe disease [36]. The VP1-F106L mutation of coxsackievirus B3 has been found to produce a rapidly replicating phenotype, accelerating the release of viral genome, whereas reducing the stability of viral capsid [37]. In addition, previous studies verified that KREMEN1 was a host entry receptor for CV-A12 [38, 39]; however, many studies focused on the host binding and entry mechanism of EV-A71 and other common enterovirus serotypes. It resulted in a lack of evidence for host binding mechanism of specific uncommon serotypes such as CV-A12 [40]. In fact, there are multiple uncommon serotypes circulating worldwide, which may further the enterovirus transmission and subsequently uncommon diseases [11]. Due to little information on the uncommon enterovirus serotypes, it is difficult to understand their transmission and phylogenetics. It warrants a need to further explore the pathogenesis of those uncommon enterovirus serotypes and determine the influence associated with potential variations.

In our study, a search of CV-A12 sequences available in the GenBank database (as of January 4, 2022) retrieved very limited number of CV-A12 sequences, as compared to other enterovirus common serotypes in both full-length and partial sequences. Furthermore, most of the CV-A12 sequences were isolated in China. It may be due to the high prevalence of EV-A in Asia [41]. Moreover, China has established a more comprehensive surveillance system for HFMD, mainly for infants and school-aged children, providing a better source for the detection and identification of uncommon serotypes. Consequently, it may result in possible bias when preparing the datasets of sequences for analysis in our study. In addition, the phylogenetic distance between the CV-A12 recently isolated in China and the CV-A12 prototype strain isolated in 1948 in the United States might affect the phylogenetic analysis and challenge the present serotyping criteria [29].

## Conclusion

This study characterized a CV-A12 strain isolated in a child with HFMD. It was phylogenetically related to multiple CV-A12 strains isolated in China during 2015–2018, whereas distant to the CV-A12 prototype strain in 1948, suggesting a continual spread in China. Furthermore, it had conserved P1, whereas divergent P2 and P3 regions. A recombination with a CV-A5 strain was identified in P2, indicating the extensive recombination among EV-A serotypes. It warrants strengthening the routine surveillance for uncommon enterovirus serotypes, particularly on possible recombination and variation.

## Supplementary Information

**Additional file 1**. Supplementary tables showing the information of sequences analyzed. 

## Data Availability

The CV-A12 and other EV-A sequences used in this study are available in GenBank of the National Center for Biotechnological Information (https://www.ncbi.nlm.nih.gov/genbank/). The accession numbers of all sequences are showed in Additional file 1: Tables S1–S3.

## Abbreviations

BEAST: Bayesian evolutionary analysis sampling trees
CDC: Center for Disease Control and Prevention
CDS: Coding sequence
CV-A12: Coxsackievirus A12
EV: Enterovirus
EV-A: Enterovirus A
HFMD: Hand, foot, and mouth disease
MCC: Maximum clade credibility
MEGA: Molecular Evolutionary Genetics Analysis
qPCR: Real-time polymerase chain reaction
RT-PCR: Reverse transcription polymerase chain reaction
