# Supplementary material for: Molecular characteristics of a coxsackievirus A12 strain in Zhejiang of China, 2019
Source: Virol J. 2022 Oct 12;19:160. doi: 10.1186/s12985-022-01892-1 (PMC9555000; doi:10.1186/s12985-022-01892-1)
Supplement: Supplementary file 1 — Additional file 1. Supplementary tables showing the information of sequences analyzed. [file 12985_2022_1892_MOESM1_ESM.docx]

**Supplementary Table 1** Dataset 1 of complete coding sequences (CDS) of coxsackievirus A12 (CV-A12) and other enterovirus A (EV-A) prototype strains in GenBank

| **Serotype** | **Accession** | **Collected region** | **Collected year** | **Strain** |
| --- | --- | --- | --- | --- |
| CV-A12 | AY421768* | United States | 1948 | Texas-12 |
|  | KF422142 | China | 2009 | QD-LXH535/SD/CHN/2009 |
|  | KF422143 | China | 2011 | QD-HDH507/SD/CHN/2011 |
|  | MH888020 | China | 2018 | HEV786663 |
|  | MK061424 | China | 2018 | HEV16537821CA12 |
|  | MK061425 | China | 2018 | HEV16087564CA12 |
|  | MK977592 | China | 2015 | D89 |
|  | MK977588 | China | 2015 | 278 |
|  | MK977587 | China | 2015 | 274 |
|  | MK977591 | China | 2015 | D79 |
|  | MK977593 | China | 2016 | WF57 |
|  | MK977590 | China | 2015 | 283 |
|  | MK977589 | China | 2015 | 280 |
| CV-A2 | AY421760* | United States | 1947 | Fleetwood |
|  | KP289360 | China | 2013 | CV-A2/P478/2013/China |
|  | MK967662 | China | 2015 | LW517 |
|  | MK967659 | China | 2015 | LW574 |
|  | MK967656 | China | 2015 | ZB285 |
|  | MK967664 | China | 2016 | 16034 |
|  | MK967658 | China | 2016 | LW636 |
| CV-A3 | AY421761* | United States | 1948 | Olson |
| CV-A4 | AY421762* | United States | 1948 | High Point |
|  | MF422546 | Chinese Taiwan | 2008 | 61229-836 |
|  | MN964080 | China | 2018 | CV-A4/2018/herpangina/31 |
|  | MN964082 | China | 2018 | CV-A4/2018/herpangina/16 |
|  | MN964081 | China | 2018 | CV-A4/2018/herpangina/25 |
|  | OL519574 | China | 2018 | xz009-HEV-1 |
| CV-A5 | AY421763* | United States | 1950 | Swartz |
|  | OK334538 | China | 2017 | 3490 |
|  | OK334537 | China | 2017 | 3474 |
|  | MN663160 | China | 2017 | 3487 |
|  | MW079817 | China | 2017 | 3487 |
| CV-A6 | AY421764* | United States | 1949 | Gdula |
|  | MN337601 | China | 2013 | S0164b/AH/2013 |
|  | KP289365 | China | 2013 | CV-A6/P289/2013/China |
|  | KP289366 | China | 2013 | CV-A6/P423/2013/China |
| CV-A7 | AY421765* | United States | 1949 | Parker |
| CV-A8 | AY421766* | United States | 1949 | Donovan |
|  | MT648783 | China | 2015 | HZ040/SD/CHN/2015 |
|  | KM609477 | China | 2012 | CVA8/SZ127/CHN/2012 |
|  | KM609475 | China | 2012 | CVA8/SZ93/CHN/2012 |
| CV-A10 | AY421767* | United States | 1950 | Kowalik |
|  | MF422531 | Chinese Taiwan | 2008 | 61216-2814 |
| CV-A14 | AY421769* | Republic of South Africa | 1950 | G-14 |
| CV-A16 | U05876* | Republic of South Africa | 1951 | G-10 |
| EV-A71 | U22521* | United States | 1970 | BrCr |
| EV-A76 | AY697458* | France | 1991 | FRA91-10369 |
| EV-A89 | AY697459* | Bangladesh | 2000 | BAN00-10359 |
| EV-A90 | AY697460* | Bangladesh | 1999 | BAN99-10399 |
| EVA91 | AY697461* | Bangladesh | 2000 | BAN00-10406 |
| EVA92** | EF667344* | United States | 2007 | RJG7 |
| EVA114 | KU355876* | India | 2013 | V13-0285 |
| EVA121 | KU355877* | India | 2013 | V13-0682 |
| EVA123** | AF326761* | United States | 1956 | OM112t(P12) |
| EVA124** | AF326764* | United States | 1964 | OM22(P15) |
| EVA125** | AF326750* | United States | 1962 | A13 |
| SV19** | AF326754* | United States | 1956 | M19s(P2) |

This dataset contained two subsets. The first subset was prototype subset, containing all EV-A serotype prototype strains and all CV-A12 strains. The second subset was BLAST subset, including all the sequences without asterisk* and the CV-A12 prototype strain Texas-12. In the study, we used the first subset for similarity analysis and the second one for recombination analysis.

* EV-A serotype prototype strain

** Simian enterovirus

**Supplementary Table 2** Dataset 2 of partial coxsackievirus A12 (CV-A12) VP1 sequences in GenBank

| **Accession** | **Collected region** | **Collected year** | **Strain** |
| --- | --- | --- | --- |
| AY421768 * | United States | 1948 | Texas-12 * |
| AB167805 | Japan | 2002 | 02-212FCR3 |
| AB162731 | Japan | 2003 | P-2172/CA12/Kanagawa/2003 |
| MH021896 | Vietnam | 2004 | PVE-1-2004 |
| JN169034 | China | 2006 | PUMCH3700Oct06 |
| JN169027 | China | 2007 | PUMCH4826Mar07 |
| JN169028 | China | 2007 | PUMCH6183Sep07 |
| JN169029 | China | 2007 | PUMCH5583Jun07 |
| JN169030 | China | 2007 | PUMCH5679Jul07 |
| KF413036 | India | 2007 | NIV078497 |
| JN169031 | China | 2008 | PUMCH7255Jun08 |
| KF422142 | China | 2009 | QD-LXH535/SD/CHN/2009 |
| JX088584 | China | 2009 | WD0029 |
| JN169032 | China | 2009 | PUMCH8592Jun09 |
| KC867079 | China | 2009 | JB143090147 |
| JN655890 | China | 2010 | M10F65 |
| JX154989 | China | 2010 | CVA12-SHZH2010-0801 |
| KF422143 | China | 2011 | QD-HDH507/SD/CHN/2011 |
| KF422145 | China | 2011 | QD-JZH622/SD/CHN/2011 |
| KF422146 | China | 2011 | QD-SFHS189/SD/CHN/2011 |
| MN187949 | China | 2011 | ZJ-EV36-2011-CVA12 |
| MN187952 | China | 2011 | ZJ-EVU20-2011-CVA12 |
| JX473470 | China | 2011 | CVA12-SHZH2011-0601 |
| JQ713869 | China | 2011 | CVA12SDCHN11105 |
| KF696708 | China | 2012 | CVA12-CS-CHN-2012-01 |
| KF661143 | Thailand | 2012 | CAV12_THA/CU277/2012 |
| KF661146 | Thailand | 2012 | CAV12_THA/CU767/2012 |
| KY865789 | Netherlands | 2013 | 3101300056_2013_CEVS |
| MK086291 | France | 2014 | CV-A12_WWTP_OTU_385 |
| MK977592 | China | 2015 | D89 |
| MK977588 | China | 2015 | 278 |
| MK977587 | China | 2015 | 274 |
| MK977591 | China | 2015 | D79 |
| MK977590 | China | 2015 | 283 |
| MK977589 | China | 2015 | 280 |
| MK086292 | France | 2015 | CV-A12_WWTP_OTU_1418 |
| MK086293 | France | 2015 | CV-A12_WWTP_OTU_1432 |
| MK977593 | China | 2016 | WF57 |
| MT394612 | China | 2017 | NPEV/CA12/JX66-2/2017 |
| MH888020 | China | 2018 | HEV786663 |
| MK061424 | China | 2018 | HEV16537821CA12 |
| MK061425 | China | 2018 | HEV16087564CA12 |
| MN541005 | China | 2018 | CV-A12/SWG37/SD/CHN/2018 |
| **OM638431 **** | **China** | **2019** | **PJ201984 **** |

* EV-A serotype prototype strain

** PJ201984 was collected in this study

**Supplementary Table 3** Dataset 3 of full-length coxsackievirus A12 (CV-A12) VP1 sequences in GenBank

| **Accession** | **Collected region** | **Collected year** | **Strain** |
| --- | --- | --- | --- |
| AY421768 * | United States | 1948 | Texas-12 * |
| JX088584 | China: Shandong | 2009 | WD0029 |
| KF422142 | China: Shandong | 2009 | QD-LXH535/SD/CHN/2009 |
| KF422143 | China: Shandong | 2011 | QD-HDH507/SD/CHN/2011 |
| KF422144 | China: Shandong | 2011 | QD-PDHS345/SD/CHN/2011 |
| KF422145 | China: Shandong | 2011 | QD-JZH622/SD/CHN/2011 |
| KF422146 | China: Shandong | 2011 | QD-SFHS189/SD/CHN/2011 |
| KF422147 | China: Shandong | 2011 | QD-CYHS249/SD/CHN/2011 |
| KF696708 | China: Hunan | 2012 | CVA12-CS-CHN-2012-01 |
| MK977587 | China: Shandong | 2015 | 274 |
| MK977588 | China: Shandong | 2015 | 278 |
| MK977589 | China: Shandong | 2015 | 280 |
| MK977590 | China: Shandong | 2015 | 283 |
| MK977591 | China: Shandong | 2015 | D79 |
| MK977592 | China: Shandong | 2015 | D89 |
| MK977593 | China: Shandong | 2015 | WF57 |
| MH888020 | China: Yunnan | 2018 | HEV786663 |
| MK061424 | China: Yunnan | 2018 | HEV16537821CA12 |
| MK061425 | China: Yunnan | 2018 | HEV16087564CA12 |
| MN541005 | China: Shandong | 2018 | CV-A12/SWG37/SD/CHN/2018 |
| MT495411 | China: Yunnan | 2019 | P6-CV-A12-HH-YN-2019 |
| MT495412 | China: Yunnan | 2019 | P7-CV-A12-HH-YN-2019 |
| **OM638431 **** | **China: Zhejiang** | **2019** | **PJ201984 **** |

* EV-A serotype prototype strain

** PJ201984 was collected in this study
